# How medical technologies shape the experience of illness

**DOI:** 10.1186/s40504-018-0069-y

**Published:** 2018-02-03

**Authors:** Bjørn Hofmann, Fredrik Svenaeus

**Affiliations:** 1 Institute for the health sciences, Norwegian University of Science and Technology (NTNU), Gjøvik, Norway; 2 Centre of Medical Ethics, University of Oslo, PO Box 1130, Blindern, N-0318 Oslo, Norway; 3 Centre for Studies in Practical Knowledge, School of Culture and Learning, Södertörn University, 141 89 Huddinge, Sweden

**Keywords:** Illness, Technology, Disease, Phenomenology

## Abstract

In this article we explore how diagnostic and therapeutic technologies shape the lived experiences of illness for patients. By analysing a wide range of examples, we identify six ways that technology can (trans)form the experience of illness (and health). First, technology may create awareness of disease by revealing asymptomatic signs or markers (imaging techniques, blood tests). Second, the technology can reveal risk factors for developing diseases (e.g., high blood pressure or genetic tests that reveal risks of falling ill in the future). Third, the technology can affect and change an already present illness experience (e.g., the way blood sugar measurement affects the perceived symptoms of diabetes). Fourth, therapeutic technologies may redefine our experiences of a certain condition as diseased rather than unfortunate (e.g. assisted reproductive technologies or symptom based diagnoses in psychiatry). Fifth, technology influences illness experiences through altering social-cultural norms and values regarding various diagnoses. Sixth, technology influences and changes our experiences of being healthy in contrast and relation to being diseased and ill. This typology of how technology forms illness and related conditions calls for reflection regarding the phenomenology of technology and health. How are medical technologies and their outcomes perceived and understood by patients? The phenomenological way of approaching illness as a lived, bodily being-in-the-world is an important approach for better understanding and evaluating the effects that medical technologies may have on our health, not only in defining, diagnosing, or treating diseases, but also in making us feel more vulnerable and less healthy in different regards.

## Introduction

Medical technologies strongly influence not only the way doctors encounter and treat patients but also how they understand their ailments and complaints. This is also true regarding the patients themselves: the new technologies affect the way patients think about and perceive their health condition. Moreover, the effects of medical technologies are not limited to what occurs in hospitals or health care centres, the effects are also the result of how information about new diagnostic and therapeutic possibilities is disseminated via traditional and social media and, also, sold and used on direct consumer basis (diagnostic tests).

This paper investigates in some detail how medical technologies shape the experience of illness and related conditions. Medical technology is understood in a broad manner including diagnostic as well as therapeutic technologies. One way in which therapeutic technologies may affect illness experiences is to relieve or eliminate symptoms. However, as we will see, the ways in which technologies may transform experiences of illness are much richer than this. Sometimes technologies even appear to create illness when persons refocus their attention on experiences they had heretofore considered nonsignificant.

Previously it has been argued that technology impacts the way diseases are defined and diagnosed (Hofmann, 2013). Here we will argue that technology not only influence the way diseases are specified, but also how we experience illness and health. This is a significant step since it underlines that medical technology is not only transforming the way we specify and treat dysfunctions of human bodies, it also transform the way persons *experience* their bodily condition. The overarching term we have chosen to consider this transformative effect technologies may have on illness experience is “shaping.” As we will see, medical technologies can shape (form) illness experiences in many ways when they provide focus and patterns for persons’ self-understanding.

Technologies may affect the way diseases are diagnosed by way of lab tests and imaging procedures that *establish* the presence of bodily dysfunctions, or even, some will claim, *create* the diseases as a result of the testing procedure. Debates between realists and constructivists in the philosophy of medicine and technology have been fierce and are hard to settle. However, much less has been written about how technology influences persons’ *experiences* of the state of their bodies when they are diagnosed. Illness is a basic human experience, and as other things in human life, it may be subject to influence by technological interventions. Illness is different from disease since it relies on the first-person perspective of bodily suffering – not only having, but also existing as a body – in contrast to the third-person perspective of investigating this body as a biological organism (Carel, 2016). In such a phenomenological understanding, the suffering person is said to experience illness as and through her *lived* bodily being-in-the-world, in contrasts to the living body studied by medical science and technologies (Leder, 2016). The lived, experienced body is made possible by a living, biological body, and technology may affect this lived bodily experience either through the ways it changes biological functionality and/or through the way it is perceived by the person existing as a lived bodily being-in-the-world (Svenaeus, 2017).

Accordingly, the objective of this study is to investigate how illness is formed by technology, i.e., how our experiences of our bodies are shaped by the application of technologies in health care. By studying a series of examples, we have developed a typology of how illness experience can be shaped by technology. We then use this typology to reflect on the phenomenology of medical technology and health. The phenomenological way of approaching illness as a lived, bodily being-in-the-world is an important approach for better understanding and evaluating the effects that medical technologies may have on our health, not only in diagnosing or treating diseases, but also in making us feel more vulnerable and less healthy in different regards.

## Technology shaping the experience of illness: Towards a typology

### Revealing underlying disease

One way that the application of medical technology may lead to an experience of illness is by revealing asymptomatic signs or markers and bringing them to a person’s attention. Imaging examinations, blood tests or genetic tests may disclose (anatomical, biochemical, immunological, biomolecular or other) conditions that influence the person’s conception of his or her health and wellbeing – even though he or she does not (yet) suffer from any symptoms of the disease. That is: various diagnostic tests may reveal that a person is diseased although she does not have any symptoms or experiences of this beyond having a positive test result.

A common example of this would be the incidental finding of a disease during a check-up, a routine test, or a direct-to-consumer test taken for fun. This could for example be a pap smear test to detect cervical cancer (Forss, 2007). Here the tests may alter persons’ conception of their health and make them feel ill. Discovering a tumour on a routine scan may generate worries, existential angst, and even pain (Walker and Rogers, 2017, Pickering, 2006, Petersen et al., 2010). Awareness of diseases, such as hypothyroidism, Diabetes Mellitus, and hypertension has been shown to be associated with poor perceived health (Jørgensen et al., 2015), suggesting that diagnostic labelling through technological measurements can have a negative effect on self-rated health and illness experience. A further example of this would be a genetic test for Huntington’s Disease (HD) where a positive result may change the person’s experience of his health significantly despite the disease still being dormant (Timman et al., 2004). As a matter of fact, such a test can completely change the self-conception, prospect of life, family planning, and relationship with relatives of a person. The point is that by making persons aware of a disease the technology alters the persons’ conception of their bodies and themselves. Hence, technology can *create illness* when persons experience their bodies and lives in new ways.

### Revealing risk of disease

A related, but somewhat different, way that technology can give rise to experiences related to illness is by revealing risk factors for developing diseases. In a way, the genetic test for HD is an example of this, but in this case the risk of falling ill is 100% whereas in most cases the risk of developing the disease in question will be significantly lower. Measuring high blood pressure, high cholesterol level, or testing positive for a genetic test that is related to disease risk can alter one’s conception of oneself and one’s body as vulnerable and at risk of falling ill. One example is Bone density measurements (DXA) which have altered people’s behaviour, experience of anxiety, and feeling of being vulnerable (Rimes et al., 1999). Another example is Long QT Syndrome (LQTS) where ECG and genetic tests have defined and formed the conception of as well as affected people’s experience of risk, vulnerability, and illness (Hendriks et al., 2008, Andersen et al., 2008). In particular, positive ECG test together with a specific genetic test result may give a risk score which alters a person’s conception of health status, spur behavioural changes, and warrant preventive treatment.

Here the point is that technology alters the person’s conception of themselves and their vulnerability as well as their behaviour by revealing a disease risk. Hence, technology *creates anxiety and vulnerability awareness*. This is closely related to risk aversion and a more risk-oriented conception of disease (Aronowitz, 2009, Aronowitz, 2015).

### Affecting illness experience

Technology can also transform already present illness experiences in various ways. First, the technology and its applications may become *part* of or even *replace* the experience of illness. One example is how blood sugar measurements (HgA1C) alters the experience of (living with) diabetes (Hilden, 2002, Mol, 2000). The practice of glucose measurements and reading, interpreting, and handling the test results become part of the illness experience. This can also be seen in continuous glucose monitoring, illustrated by the following quote:

“I wish I had known the data would be addictive at first. The first time I wore a Dexcom sensor, it was back in 2006 and was one of the first marketed versions of the system. But I was hooked on the data. I looked at the receiver every five minutes and went bonkers trying to make sense of the trends. The trouble was that the readings were far less accurate back on the Dexcom STS, but I took them as seriously as the numbers on my glucose meter. For the first few weeks of wearing the Dexcom, I drowned in data, obsessively checking it and chasing slight blood sugar climbs with aggressive correction boluses. I needed to learn to let the data flow into my management, not change the flow of my management.”(Sparling, 2014)

Second, technology changes *bodily self-conception* in illness. Imaging techniques, such as X-ray, Computer Tomography (CT), and Magnetic Resonance Tomography (MRT), have changed our conception of our bodies making us experience them in new ways. The patient’s illness experience is changed as when “popular experience is overtaken by technical expertise, including complex organizations of treatment” (Frank, 2013). E.g. a soccer-player might state that he has some pain in his meniscus or a patient can feel his “large intestines a bit bound” based on seeing X-ray images only (Nessa and Malterud, 1998). As pointed out by Gerrits about assisted reproductive technologies:

“When people make use of medically assisted conception, they come to view their bodily functioning and conception differently. They come to see conception as a process that is split up into many small steps, which can be followed and ‘seen’ by means of visualizing medical technologies, namely the microscope, the ultrasound, the X-Ray, the laparoscopy, the hysteroscopy, and further laboratory technologies.” (Gerrits, 2008)(p.225)^1^

Medical technology makes patients experience their bodies in certain ways, and the authority of the technology rests primarily on its ability to “create a straightforward sense of reality and visual pleasure” (Georges, 1996). Accordingly, people have become more inclined to assess their life situation with medical criteria (Gerrits, 2008)(p. 228). Correspondingly, neuromodulation technology, such as spinal cord stimulation, represents an embodiment and incorporation as “bodies and the way they are experienced are reconfigured” (Dalibert, 2016).

Third, technology may constitute or influence the *reliability* of experienced symptoms when *confronted* with measurement results. For instance, the experience of sweating, dizziness, headache, or shortness of breath may be influenced by various measurements when the subjective experience of the patient is projected onto para-clinical signs and tests (Hofmann, 2002). An example of this is the way patients with renal failure in haemodialysis rely on measurements of their blood pressure and dry weight and reinterpret and change their experiences of dizziness or pain in response to the numbers (Gunnarson, 2016)(p.207–211).

Fourth, it is not only diagnostic technologies that transform illness experiences. *Drugs* such as methylphenidate has not only contributed to define ADHD as a disorder, but also the experience of living with ADHD (when being treated with the medication). Correspondingly, bariatric surgery has altered the conception of obesity from being weakness of the will to become a metabolic condition which can easily be treated (Hofmann, 2010). This conceptual change influences and alters people’s experience of obesity.

The point regarding how technologies may mediate and transform illness experiences in these four ways is that technology can *change symptom formation* through the entities and numbers measured and shown by the technologies that we use to manage disease.

### Technological medicalization

Technologies may affect or redefine our experiences of certain ordinary life experiences as diseased or disordered rather than normal but unfortunate. This, in turn, may lead to experiences of alienation and discomfort. The most obvious example of this is the potential medicalization process of contemporary diagnostic psychiatry. A patient who is feeling restless and worried, having difficulties to relax, concentrate, finding focus in life and sleeping at night may change the view on her condition when told that although the doctor cannot find anything physically wrong with her she is probably suffering from an anxiety disorder, which can be treated by cognitive behavioural therapy and SSRI’s (Svenaeus, 2013). The doctor will motivate this claim by using and referring to diagnostic questionnaires and test scores identifying and estimating the significance of various symptoms such as the ones mentioned above. If the patient did not consider herself ill previous to receiving the diagnosis, the diagnostic manual (DSM or ICD) could be said to have *created* her illness in such cases (Horwitz, 2002).

New mental disorders such as Hoarding Disorder and Disruptive Mood Dysregulation Disorder (listed in DSM-5, 2013) and the widening of diagnostic labels such as depression bipolar disorder, autism, and ADHD are all made possible by way of diagnostic technologies, such as the DSM and ICD manuals and the diagnostic questionnaires and test scores made in sync with the manuals. Symptom based diagnostic tests not only change the experience of illness (see above), they may transform unhappiness and/or socially unaccepted behavior into a medically interpreted illness experience.

There are also examples within somatic medicine of how medical technologies can transform experiences of unhappiness and dissatisfaction into experiences of being ill. For example, assistive reproductive technologies have redefined the experience of childlessness from being faith or bad luck to be something to alter and treat by making it a disease (infertility) (Mills, 2011). Correspondingly, the experience of menopause has been altered by pharmaceutical treatment (Houck, 2005, Santos, 1997). Technology has also contributed to transform what has been looked upon as the natural processes of ageing and senescence into an explanatory model based on pathological processes. As argued by Armstrong:

“If its [the chronic disease of aging] disabling impact was one of its defining characteristics this could not easily be identified or assessed using the usual tools of clinical medicine such as a stethoscope, a blood test or an X-ray. Instead new technologies emerged to capture this novel medical construct and, given the population framework of this new medical object, it propelled epidemiology and public health from their former sanitary realm to engage with these new illnesses/diseases with disabling attributes.” (Armstrong, 2014).

This redefinition of experiences of ordinary life phenomena can also be seen in so-called enhancement technologies, which alter and blur traditional distinctions between natural and artificial, between therapy and enhancement, between health and disease, and between arbitrary events and responsible actions (Hofmann, 2017). In the same manner as vaccines have altered our conceptions and experiences of, as well as responsibilities for, previously ordinary life experiences, such as having measles, mumps, and rubella, we now have a plethora of new technologies altering our conception of what it is to be a human being (Sharon, 2013, Dalibert, 2014) as well as our related responsibilities. As we have the possibility to enhance humans’ characteristics (such as intelligence), we gain a responsibility to do so (Savulescu, 2005, Savulescu and Kahane, 2009, Moen, 2016), drawing attention to this characteristic, and making those that are not enhanced feel inferior. Technological possibilities tend to move physical conditions from the realm of fate to the realm of human intervention and responsibility, fuelling a medicalized framing of ourselves.

The point here is that technology may make *new phenomena and areas of ordinary life* subject to measurement, attention, and medical interpretation.

### Technology and the social-cultural roles of diagnoses

Additionally, technology may shape illness experiences through social norms and values fostered by various technologies. In a seminal article, Conrad and Barker point out how illness is socially constructed in three ways: First, some illnesses are particularly embedded with cultural meaning which shapes how society responds to those afflicted and influences the experience of the illness. Second, all illnesses are socially influenced at the experiential level, based on how individuals come to understand and live with their illness. Third, medical knowledge about illness and underlying diseases is not necessarily given by nature but is constructed and developed by a range of stakeholders (Conrad and Barker, 2010). Technology seems to play a role in all three cases.

One example of technology’s influence on social norms is through altering the status and prestige of a particular disease. Technology affects the (professional) prestige of diseases and specialties (Album et al., 2017, Album and Westin, 2008). The use of advanced technologies renders the disease high prestige in contrast to illness experiences of which there is no known cause. This tends to influence the experience of having a certain disease, e.g., myocardial infarction versus fibromyalgia. Accordingly, “contested illness sufferers are burdened by the cultural meaning of a medically invisible condition in an era of high-tech biomedicine ” (Conrad and Barker, 2010).

A related example of technology’s influence in this domain is through inducing or relieving social stigma. Various diseases have different stigma, which strongly influences illness experience (Garand et al., 2009, Herek et al., 2003). Technology may alter this by increasing or decreasing stigma. Genetic tests have moved obesity from being a moral disease (weakness of the will) to a genetic disease, and bariatric surgery has made it a surgical or metabolic disease – altering both its status, prestige, and stigma. Yet an example is how social media technology strongly has influenced the social construction of inflammatory bowel disease (Frohlich, 2016).

One social aspect of technologies is that it moves a condition from the realm of chance and fate to that of control and responsibility. Conditions previously conceived of as fate that needs to be faced become diseases experienced as illness as soon as they can be anticipated with diagnostic technologies or treated with technological interventions. One example is prenatal ultrasound altering conceptions of one’s own body, pregnancy, and being expecting (including moral dilemmas of abortion) (Verbeek, 2008).

The point here is that technology shapes our illness experience through *the social norms and values fostered by technology*.

### Medical technologies and the focus on health

A special case of how medical technologies affect norms regarding status and prestige of different human behaviours and conditions is the way they have transformed our experience of the opposite of illness: health. Health is increasingly becoming not only a condition that makes possible to realize various human projects and goods, but a life goal in itself that needs to be managed and controlled (Conrad, 2007). New mobile apps and a range of wearables mediate the conception of bodily activity, sleep, nutrition, digestion, etc. through measurements of bodily functions and make these and other ordinary human experiences subject to medical attention. Medical technologies scrutinize the functions of our body and provide information not only about asymptomatic diseases or risks for developing diseases (1. and 2.) but also about how stable and strong our health is. It thereby directs our attention toward the usually “silent” dimension of lived embodiment (Gadamer, 1996), which can be enjoyed but also worried about for the reason of not being good enough according to standards provided by way of or in association with the measurement technologies (Sharon and Zandbergen, 2017).

Accordingly, in addition to affecting illness experiences (3.) medical technologies also affect the experience of health and this, in a way, is also a case of medicalization (healthization) by way of technology (4.). Digital self-tracking by way of health apps or wearables is also, as already mentioned, related to the way technologies can change the social-cultural value and prestige associated with a disease (5.), because it leads to an increased value being associated with health itself. Rather than being something that we take for granted, health is now targeted and mediated by way of medical technologies and thereby standardly subjected to various regimes in which we change our life style and preferences in order to stay healthy or even becoming *more* healthy (meaning having better numbers as regards the bodily functions that we measure).

Altogether, we have elaborated a typology based on how technology shapes illness. In particular, we have found that:

1. Technology may create illness by making persons experience their bodies and lives in new ways, e.g., by revealing underlying disease.
2. Technology may alter persons’ conception of themselves and their vulnerability as well as their behaviour by revealing disease risks.
3. Technology can modify already present illness experience in several ways. We have mentioned four such ways.
4. Technology can shape illness experience by making new phenomena and areas of ordinary life subject to measurement, attention, medical interpretation, and management.
5. Technology influences illness experiences through altering social-cultural norms and values regarding various diagnoses as well as moving experiences from the realm of chance and fate to that of control and responsibility.
6. Technology shapes our experiences of illness through its measures to monitor and measure our health.

Hence, our illness experience is shaped by technology in a number of ways. In the same manner as Wardrope underscores that “the interpretation of human experience overwhelmingly [is made] in medical terms” (Wardrope, 2017) we find that *the experience of illness overwhelmingly is made in and through technological terms*. Technology has “an impact on how patients experience the disease as a part of their lives” (Kiran et al., 2015).

## Illness and the philosophy of technology

This study is by no means exhaustive. Other examples could have been explored. The typology is by no means fixed either. Slightly different categories could have been used, as they are interrelated and partly overlapping. Nonetheless, we believe they make it clear how illness experience is formed by technology in a variety of ways. Hence, it is not only *disease*, but also *illness* that is shaped by technology. In addition to the term “shape”, we have used other verbs, such as “mediate”, “affect”, “influence”, “form,” “transform”, and sometimes even “create” in describing the impact of technology on illness. Does the polysemy of these terms reveal that we have not been precise enough or are even confused about what kind of relationship holds between technology and illness? We think not. Rather the polysemy in question reveals ontological and epistemological controversies regarding the status of technology that cannot be developed in any detail or settled within the scope of a single paper, but which we would like to discuss briefly in this section before stating our conclusion.

The differences between the terms “shaping”, “mediating”, “affecting”, “influencing”, “transforming” and “creating” mirror differences in the way medical researchers, philosophers and researchers in the social and behavioural sciences view the ontological impact of modern technology. A traditional approach to technology in the analytical tradition of philosophy is that technologies make it possible to test scientific theories about the world and that they can be applied to achieve goals that humans find it worth to pursue (such as eradicating diseases) (Dusek, 2006). What technology could achieve from such a perspective is rather to influence our illness experiences by way of providing knowledge about how our body works, than to create diseases that give rise to symptoms (from this perspective symptoms that do not originate from bodily dysfunctions are not real illness experiences in the first place) (Topol, 2015). On the other side of the spectrum, we find researchers in the Science and Technology Studies (STS) tradition who claim that technologies do indeed shape our world (including human bodies) in ways that could be understood in terms of creation (Fuller, 2006). Scientific technologies make things in the world appear that would never have existed without them (e.g., elementary particles, DNA, or high blood pressure).

The phenomenological tradition, including so called post-phenomenology, could be understood as providing a middle ground between these extremes (Ihde, 2010, Rosenberger and Verbeek, 2015). The seminal text of phenomenological technology studies is Martin Heidegger’s essay “The Question Concerning Technology”, published in 1954, in which the author famously claims that the essence of modern technology is that it makes (human) nature appear as a resource (Heidegger, 1977). The modern hydroelectric power station straddling the river Rhine is Heidegger’s main example in the essay. The power plant makes the river a ‘water-power supplier’ in contrast to the old wooden bridge over the Rhine, which allows the river to be a river and not just a source of energy, according to Heidegger (Heidegger, 1977).

Heidegger says explicitly in the essay that the essence of technology he is trying to articulate is not the instrumental use of a piece of technology in the sense of goals that might be different depending upon the occasion. He finds the idea that human beings could simply choose to do whatever they want with new technological inventions a bit naïve, and in this we think that many contemporary historians and philosophers of technology would give him right. New technologies not only open up new spaces of possibilities for our doings; they also make us see things in new ways, they *shape* our experiences, dominate the *goals* of human projects, changing our views on what is worth pursuing in the first place (Svenaeus, 2017).

Such an analysis is strikingly fitting to the ways we have found medical technologies to shape illness experiences in this paper. The technologies do neither make new things appear out of the blue, nor is the influence to be characterised in the manner of discovery or treatment of diseases only, rather the technologies mediate and transform already present embodied experiences in making them appear in a more medical-scientific light invoking certain feelings, thoughts, and actions for affected persons. Moreover, technologies supply numbers and images that are not only relevant in terms of detecting disease risks, but also in providing life meaning: health and bodily strength become *life goals as such* and not only means to pursue other projects (Conrad, 2007, Vogt et al., 2016). In these ways medical technologies shape illness and health experiences and such shaping clearly has various ethical and political implications to be further explored.

## Conclusion

In this article we have explored how diagnostic and therapeutic technologies form the lived experience of illness. By analysing a wide range of examples, we have aimed to understand and systematize how medical technologies shape illness. We have identified six ways that technology can have such impact. First, technology may create awareness of disease by revealing asymptomatic signs or markers (imaging techniques, blood tests). Second, the technology can reveal risk factors for developing diseases (e.g., high blood pressure or genetic tests that reveal risks of falling ill in the future). Third, the technology can affect and change an already present illness experience in several ways (e.g., the way blood sugar measurement affects the perceived symptoms of diabetes). Fourth, therapeutic technologies may redefine our experiences of a certain condition as diseased rather than unfortunate (e.g. assisted reproductive technologies or symptom based diagnoses in psychiatry). Fifth, technology influences illness experiences through altering social-cultural norms and values regarding various diagnoses. Sixth, technology influences and changes our experiences of being healthy in contrast and relation to being diseased and ill. The phenomenological way of approaching illness as a lived, bodily being-in-the-world is an important approach for better understanding and evaluating the effects that (medical) technologies may have on our health, not only in diagnosing or treating diseases, but also in making us feel more vulnerable and less healthy in different regards.

## Footnotes

1 It may be argued that pregnancy is not a disease and therefore does not give rise to any illness experience. To enter this debate is beyond the scope of this article. The point here is that the experience of the condition, which to a large extent is conceived of in medical terms, is strongly shaped by technological means (Mills, 2013, Verbeek 2008).

## References

[CR1] Album D, Johannessen LE, Rasmussen EB. Stability and change in disease prestige: a comparative analysis of three surveys spanning a quarter of a century. Soc Sci Med. 2017;180:45-51. 10.1016/j.socscimed.2017.03.020 28319909

[CR2] Album D, Westin S (2008). Do diseases have a prestige hierarchy? A survey among physicians and medical students. Soc Sci Med.

[CR3] Andersen J, Øyen N, Bjorvatn C, Gjengedal E (2008). Living with long QT syndrome: a qualitative study of coping with increased risk of sudden cardiac death. J Genet Couns.

[CR4] Armstrong D (2014). Chronic illness: a revisionist account. Sociol Health Illn.

[CR5] Aronowitz R (2015). Risky medicine: our quest to cure fear and uncertainty.

[CR6] Aronowitz RA (2009). The converged experience of risk and disease. Milbank Q.

[CR7] Carel H (2016). The phenomenology of illness.

[CR8] Conrad P (2007). The medicalization of society: on the transformation of human conditions into treatable disorders.

[CR9] Conrad P, Barker KK (2010). The social construction of illness: key insights and policy implications. J Health Soc Behav.

[CR10] Dalibert L (2014). Posthumanism and somatechnologies: exploring the intimate relations between humans and technologies.

[CR11] Dalibert L (2016). Living with spinal cord stimulation: doing embodiment and incorporation. Sci Technol Hum Values.

[CR12] Dusek V (2006). Philosophy of technology: an introduction.

[CR13] Forss A, Olin Lauritzen S, Hydén L-C (2007). What's in a pap smear? Biology, culture, technology, and self in the cytology laboratory. Medical technologies and the life world: the social construction of normality.

[CR14] Frank AW (2013). The wounded storyteller: body, illness, and ethics.

[CR15] Frohlich DO. The social construction of inflammatory bowel disease using social media technologies. Health Commun. 2016:1–9. 10.1080/10410236.2015.1077690 27050670

[CR16] Fuller S (2006). The philosophy of science and technology studies.

[CR17] Gadamer H-G (1996). The enigma of health: the art of healing in a scientific age.

[CR18] Garand L, Lingler JH, Conner KO, Dew MA (2009). Diagnostic labels, stigma, and participation in research related to dementia and mild cognitive impairment. Res Gerontol Nurs.

[CR19] Georges E (1996). Fetal ultrasound imaging and the production of authoritative knowledge in Greece. Med Anthropol Q.

[CR20] Gerrits GJE (2008). Clinical encounters: dynamics of patient-centred practices in a Dutch fertility clinic.

[CR21] Gunnarson M. Please be patient: a cultural phenomenological study of haemodialysis and kidney transplantation care: Lund Studies in Arts and Cultural Sciences; 2016.

[CR22] Heidegger M (1977). The question concerning technology, and other essays.

[CR23] Hendriks KS, Hendriks MM, Birnie E, Grosfeld FJ, Wilde AA, van den Bout J, Smets EM, van Tintelen JP, ten Kroode HF, van Langen IM (2008). Familial disease with a risk of sudden death: a longitudinal study of the psychological consequences of predictive testing for long QT syndrome. Heart Rhythm.

[CR24] Herek GM, Capitanio JP, Widaman KF (2003). Stigma, social risk, and health policy: public attitudes toward HIV surveillance policies and the social construction of illness. Health Psychol.

[CR25] Hilden PK (2002). Technology and culture--HbA1c, self care and type 1 diabetes. Tidsskr Nor Laegeforen.

[CR26] Hofmann B (2002). The technological invention of disease - on disease, technology and values.

[CR27] Hofmann B (2010). Stuck in the middle: the many moral challenges with bariatric surgery. Am J Bioeth.

[CR28] Hofmann B (2017). Limits to human enhancement: nature, disease, therapy or betterment. BMC Meical Ethics.

[CR29] Hofmann BM (2013). Technological invention of disease. Encyclopedia of creativity, invention, innovation and entrepreneurship.

[CR30] Horwitz AV (2002). *Creating Mental Illness,* Chicago.

[CR31] Houck JA. The Medicalization of menopause in America.In: Kleinman DL, Kinchy AJ, Handelsman J, editors. Controversies in Science and Technology: From Maize to Menopause. 1: Univ of Wisconsin Press. 2005;198-218.

[CR32] IhdE D (2010). Heidegger's technologies: Postphenomenological perspectives.

[CR33] Jørgensen P, Langhammer A, Krokstad S, Forsmo S (2015). Diagnostic labelling influences self-rated health. A prospective cohort study: the HUNT study, Norway. Fam Pract.

[CR34] Kiran AH, Oudshoorn N, Verbeek P-P (2015). Beyond checklists: toward an ethical-constructive technology assessment. J Responsible Innov.

[CR35] Leder D (2016). The distressed body: rethinking illness, imprisonment, and healing.

[CR36] Mills C. Futures of reproduction: bioethics and biopolitics. Dordrecht: Springer Science & Business Media; 2011.

[CR37] Moen OM (2016). Bright new world. Camb Q Healthc Ethics.

[CR38] Mol A (2000). What diagnostic devices do: the case of blood sugar measurement. Theor Med Bioeth.

[CR39] Nessa J, Malterud K (1998). “feeling your large intestines a bit bound”: clinical interaction--talk and gaze. Scand J Prim Health Care.

[CR40] Petersen A, Davis M, Fraser S, Lindsay J. Healthy living and citizenship: an overview. Critical Public Health. 2010;20(4):391-400.

[CR41] Pickering TG (2006). Now we are sick: labeling and hypertension. J Clin Hypertens.

[CR42] Rimes KA, Salkovskis PM, Shipman J (1999). Psychological and behavioural effects of bone density screening for osteoporosis. Psychol Health.

[CR43] Rosenberger R, Verbeek P-P (2015). A field guide to postphenomenology. Postphenomenological investigations: Essays on human-technology relations.

[CR44] Santos JRMD (1997). Medicalization of menopause and public health. J Psychosom Obstet Gynecol.

[CR45] Savulescu J (2005). New breeds of humans: the moral obligation to enhance. Reprod BioMed Online.

[CR46] Savulescu J, Kahane G (2009). The moral obligation to create children with the best chance of the best life. Bioethics.

[CR47] Sharon T. Human nature in an age of biotechnology: the case for mediated posthumanism. Dordrecht: Springer; 2013.

[CR48] Sharon T, Zandbergen D (2017). From data fetishism to quantifying selves: self-tracking practices and the other values of data. New Media Soc.

[CR49] Sparling KM (2014). If I knew then: continuous glucose monitoring - Dexcom [online].

[CR50] Svenaeus F. Homo patologicus: Medicinska diagnoser i vår tid: Tankekraft förlag; 2013.

[CR51] Svenaeus F (2017). Phenomenological bioethics: medical technologies, human suffering, and the meaning of being alive.

[CR52] Timman R, Roos R, Maat-Kievit A, Tibben A (2004). Adverse effects of predictive testing for Huntington disease underestimated: long-term effects 7-10 years after the test. Health Psychol.

[CR53] Topol E (2015). The patient will see you now: the future of medicine is in your hands.

[CR54] Verbeek P-P (2008). Obstetric ultrasound and the technological mediation of morality: a postphenomenological analysis. Hum Stud.

[CR55] Vogt H, Hofmann B, Getz LO (2016). The new holism: P4 systems medicine and the medicalization of health and life itself. Med Health Care Philos.

[CR56] Walker MJ, Rogers WA (2017). Diagnosis, narrative identity, and asymptomatic disease. Theor Med Bioeth.

[CR57] Wardrope A (2017). Mistaking the map for the territory: what society does with medicine: comment on Medicalisation and overdiagnosis: what society does to medicine. Int J Health Policy Manag.

